
JOURNAL INFORMATION
==============================
NLM Title Abbreviation: Behav Neurol
Journal ID: Behav Neurol
NLM Journal ID: 8914585
Journal ID: BN

Behavioural Neurology

ISSN: 0953-4180
EISSN: 1875-8584
Publisher: Wiley

ARTICLE INFORMATION
==============================
PMCID: PMC5434405
PMID: 21422560
DOI: 10.3233/BEN-2010-0300
Article ID: 202349
Article version: 1
Subjects: Research Article

The geometry of the Landmark Task

Toraldo Alessio *
Fiori Federico
Vanzan Serena
Dipartimento di Psicologia Università di Pavia Pavia Italy
*Alessio Toraldo: alessio.toraldo@unipv.it
Print publication date: 2010
Electronic publication date: 2011 Mar 7
Volume: 23
Issue: 4
First page: 221
Last page: 223
Received 2011 Jan 31; Accepted 2011 Jan 31
Copyright: Copyright © 2010 Hindawi Publishing Corporation and the authors.
Copyright year: 2010
License: This is an open access article distributed under the Creative Commons Attribution License, which permits unrestricted use, distribution, and reproduction in any medium, provided the original work is properly cited.
License URL: https://creativecommons.org/licenses/by/3.0/

==============================
Behavioural Neurology 23 (2010) 221–223
DOI 10.3233/BEN-2010-0300
IOS Press

### The geometry of the Landmark Task

Alessio Toraldo*, Federico Fiori and Serena Vanzan
*Dipartimento di Psicologia, Università di Pavia, Pavia, Italy*

### 1. Introduction

In the Landmark Task participants are shown a horizontal line divided into two segments by a transection mark (“landmark”), and asked to compare the lengths of the two segments [3,4]. This comparison can in principle be carried out by (i) considering the stimulus as a single object and assessing whether or not it is left-right symmetrical [1], or (ii) interpreting the two segments as separate objects, mentally translating one over the other and deciding whether they are congruent (see also [2,5,6]). When stimulus lines are rectangles, which is typically the case, the two types of process cannot be easily distinguished because symmetrical stimuli are (by definition) formed by two congruent segments and non-symmetrical stimuli are formed by non-congruent segments. The purpose of this work is to separate out the contribution of symmetry processing from that of translation-congruency processing by using trapezoidal instead of rectangular shapes.

### 2. Methods

### 2.1. Participants

Overall, 46 university students (F: 37; age 24.3 ± 3.2) with normal or corrected-to-normal vision, *naïve* to the purpose of the study, volunteered in two separate experiments.

### 2.2. Stimuli

180-mm long, black lines on a white background were used. The Landmark – a 0.7-mm thick, 20-mm long vertical red line – was located centrally (half the trials) or in one of four eccentric positions (−7, −3, +3, +7 mm). The two segments separated by the Landmark could be either rectangular (4-mm thick) or trapezoidal (2.6 to 5.4 mm thick), and were arranged in four types of configuration (see Fig. 1A), following a 2 × 2 design in which Symmetry – whether or not the stimulus was symmetrical with respect to the Landmark, and Repetition – whether or not the two segments were congruent after horizontal translation, were varied independently. Note that when the landmark was eccentric, stimuli were never properly *symmetrical* or *repeated*; we will use the terms *pseudo-symmetrical* and *pseudo-repeated* in these cases.

### 2.3. Procedure

Participants viewed a 15” 16:9 computer screen from a distance of 57 cm. All 256 stimuli were presented at screen centre in random order, with a 256 × 256 random dot mask being presented during the inter-stimulus interval. Neither a time limit nor specific instructions to respond quickly were given – indeed we were interested in the spontaneous strategies elicited by the Landmark task, without time pressure.

### 2.4. Left-Right (*LR*) Experiment

Twenty-nine participants had to decide which segment was the longer, left or right, by pressing one of two keys with the index (“left”) or middle (“right”) finger of their right hand.

### 2.5. Same-Different (*SD*) Experiment

Seventeen participants had to decide whether the two segments were equal or different in horizontal length, by pressing one of two keys with the index (“same”) or middle (“different”) finger of their right hand.

*Corresponding author. E-mail: alessio.toraldo@unipv.it.

ISSN 0953-4180/10/$27.50 © 2010 – IOS Press and the authors. All rights reserved

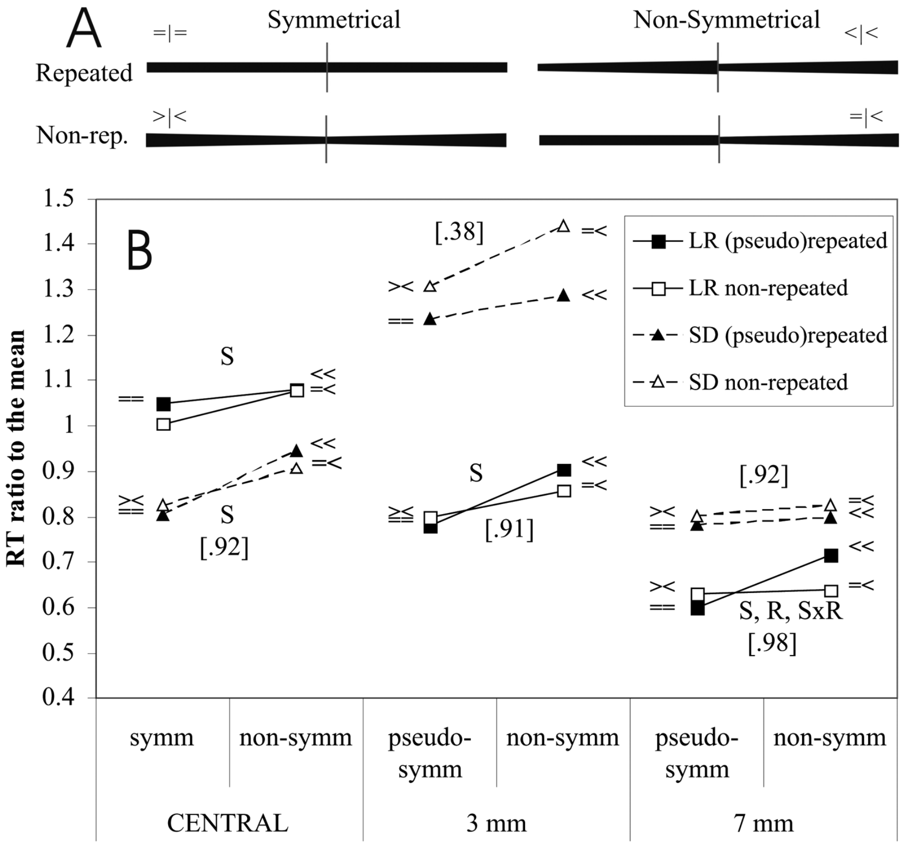

Fig. 1. **A Stimuli**. Vertical sizes are exaggerated (see text for real dimensions). Half the Symmetrical-Non Repeated stimuli had their thick ends facing inward (< | >), half outward (> | <); half the Non-symmetrical Repeated stimuli had their thick ends on the right (< | <), half on the left (> | >); the trapezoidal segment of Non-Symmetric Non-repeated stimuli was equally likely to be on either side and to face either way (= | <; = | >; < | =; > | =). **B Results**. RT (ratios to each subjects' mean RT) are reported (y axis) as a function of task and condition. LR, Left-Right task; SD, Same-different task. S, R, SxR: significant effects of Symmetry, Repetition, Symmetry X Repetition on RT ratios. Textual symbols used for the stimulus types (< | < etc) can be understood by inspecting Fig. 1A. Accuracy rates are reported in square brackets as a function of task and condition, except for central-landmark stimuli in the LR task, in which responses can neither be classified as correct nor as incorrect.

### 2.6. Measures

Accuracy and Response Times (RT) were collected. Each RT was divided by the participant's mean RT in order to control for massive inter-individual differences in RT (mean RT ranged 712–4124 ms).

### 2.7. Predictions

We predicted that, if *symmetry processing* were the main cognitive strategy involved in the Landmark Task, Symmetry would affect RT for central-landmark items. Alternatively, if *mental translation* were the main strategy, Repetition would be a crucial predictor. In either case, the effect should not appear for eccentric items, since these are neither symmetrical, nor congruent after translation.

### 3. Results

### 3.1. Accuracy

Accuracy was very high in all conditions (> 90.5%) with the only exception being the 3-mm eccentric stimuli on the SD task, which yielded a 38% hit rate. Since participants proved capable of detecting 3-mm differences (by their 90.5% accuracy in the LR task with identical stimuli), we attributed the low performance on the SD task to the use of a more conservative response criterion.

### 3.2. Response times

RT ratios decreased linearly with eccentricity on the LR task; on the SD task RT were slowest for 3-mm eccentricity, and roughly equal for central and 7-mm stimuli. These patterns were again compatible with the assumption of a more conservative response criterion applied in SD than in LR.

### *3.2.1. Same-Different task*

The pattern was fully consistent with the hypothesis of active use of symmetry, with a significant main effect of Symmetry [F(1,16) = 27.841, $p < 0.001$] on central, and no effect on eccentric landmarks [F(1,16) = 0.455, $p = 0.509$].

### *3.2.2. Left-Right task*

Symmetry was again a significant predictor for central landmarks [F(1,28) = 4.948, $p = 0.034$], but an unexpected result emerged from eccentric landmarks: a significant Symmetry X Repetition interaction [F(1,28) = 17.942, $p < 0.001$], suggesting that the $> \mid >$ category poses some additional difficulty with respect to the other three categories (see Fig. 1B). We also compared $< \mid <$ and $> \mid >$ but failed to find any significant difference in RT or accuracy.

### 4. Discussion

In the Same-Different version of the Landmark task the prediction of the *symmetry processing* hypothesis was fully met.

Data from the Left-Right task were also compatible with the symmetry processing hypothesis, in that an effect of Symmetry was found for central-landmark stimuli. However, a puzzling pattern emerged from the eccentric landmarks, i.e., a selective *dis*advantage for non-symmetrical repeated stimuli ($< \mid <$). An interpretation in terms of some form of symmetry processing would be supported by the advantage of $= \mid =$ over $< \mid <$ (see Fig. 1, bottom-right plot), but is contradicted by the absence of any such effect between $> \mid <$ and $= \mid <$, which also differ for (a)symmetry. Neither does an account in terms of mental translation hold: if anything, it would have predicted an *advantage* for $< \mid <$ over $= \mid <$; instead, a *dis*advantage was observed. We speculated that the overall geometrical structure of $< \mid <$ might suggest a double arrow pointing leftwards, and thus possibly interfere with response selection. However if this had been the case, a spatial compatibility effect would have emerged, with "left" response being facilitated in the case of $< \mid <$ and inhibited in case of $> \mid >$, and vice versa for "right" responses. However no such effect emerged. So we still lack an obvious explanation for this data configuration.

Overall, these data strongly suggest that symmetry processing, and not mental translation, is the main strategy applied by normal participants to solve the two typical versions of the Landmark Task.

### References

[1] M. Bertamini, J.D. Friedenberg and M. Kubovy, Detection of symmetry and perceptual organization: the way a lock-and-key process works, *Acta Psychol* (*Amst*) **95** (1997), 119–140.

[2] G.R. Fink, J.C. Marshall, P.H. Weiss, I. Toni and K. Zilles, Task instructions influence the cognitive strategies involved in line bisection judgements: evidence from modulated neural mechanisms revealed by fMRI, *Neuropsychologia* **40** (2002), 119–130.

[3] M. Harvey, A.D. Milner and R.C. Roberts, An investigation of hemispatial neglect using the Landmark Task, *Brain Cogn* **27** (1995), 59–78.

[4] A.D. Milner, M. Brechmann and L. Pagliarini, To halve and to halve not: an analysis of line bisection judgements in normal subjects, *Neuropsychologia* **30** (1992), 515–526.

[5] D.T. Wilkinson and P.W. Halligan, The effects of stimulus symmetry on landmark judgments in left and right visual fields, *Neuropsychologia* **40** (2002), 1045–1058.

[6] D.T. Wilkinson and P.W. Halligan, Stimulus symmetry affects the bisection of figures but not lines: evidence from event-related fMRI, *NeuroImage* **20** (2003), 1756–1764.
